# Impact of Rowing Training on Quality of Life and Physical Activity Levels in Female Breast Cancer Survivors

**DOI:** 10.3390/ijerph18137188

**Published:** 2021-07-05

**Authors:** Juan Gavala-González, Amanda Torres-Pérez, José Carlos Fernández-García

**Affiliations:** 1 Department of Physical Education and Sports, University of Seville, 41013 Seville, Spain; jgavala@us.es; 2 Researching in Sport Science: Research Group (CTS-563) of the Andalusian Research Plan, 29010 Malaga, Spain; jcfg@uma.es; 3 Department of Didactics of Languages, Arts and Sport, University of Malaga, Andalucía-Tech, IBIMA, 29010 Malaga, Spain

**Keywords:** breast cancer, rowing, exercise, quality of life, perceived health, IPAQ-SF, SF-36

## Abstract

The aim of this longitudinal study was to determine whether a rowing training program improved the quality of life and the physical activity levels in female breast cancer survivors (*n* = 28) (stage 1–4.54%; stage 2–36.36%; stage 3–54.54%; and stage 4–4.54%), diagnosed 4.68 ± 3.00 years previously, who had undergone a subsequent intervention (preservation 56.53% and total mastectomy 43.47%) and had a current mean age of 52.30 ± 3.78 years. The participants (*n* = 28) engaged in a 12-week training program, each week comprising three sessions and each session lasting 60–90 min. The short form of the International Physical Activity Questionnaire (IPAQ-SF) and the Short Form 36 Health Survey (SF-36) were also administered. The results showed statistically significant improvements in levels of physical activity and in the dimensions of quality of life. We can conclude that a 12-week rowing training program tailored to women who have had breast cancer increases physical activity levels, leading to improved health status and quality of life.

## 1. Introduction

Cancer is the second leading cause of death worldwide, representing about 9.6 million deaths in 2018, which means that one in six deaths globally is due to this disease [1,2]. In women, breast cancer is the most common cancer, affecting around 2.1 million women in 2018, i.e., one in four cancers diagnosed is breast cancer [2,3,4].

The rise in the number of cancer cases diagnosed in recent years has been associated with population growth, closely linked to increased life expectancy, and therefore with aging, considering age as a fundamental risk factor for developing cancer. It has also been related to the increase in early detection as well as improvements in primary care and in early diagnosis programs, which, although they lead to higher numbers of cases, are in turn related to a decrease in mortality [4,5,6].

One-third of diagnosed cancer cases could be prevented if exposures to various lifestyle-related risk factors were eliminated or reduced, such as smoking; consumption of harmful substances such as alcohol; an unhealthy, high-calorie diet with a high intake of saturated animal fats and sugars; and/or a sedentary lifestyle [1,4,5,7]. A large body of scientific evidence shows that physical activity has positive effects on the general population, improving health status, mood, body composition, quality of life [7,8,9,10,11], and preventing the onset of numerous diseases, including various types of cancer, such as breast cancer [12,13,14].

Physical activity has been associated with a lower risk of developing breast cancer [12,13,15], with a decrease in the probability of relapse and with a higher survival rate [13,14,15]. In individuals with cancer, physical activity has benefits for their health: reduced fatigue, improved strength levels, and improved quality of life and physical function [15,16,17,18,19].

However, despite the evidence supporting physical activity, two out of three cancer patients do not perform the minimum levels of exercise recommended by the American College of Sports Medicine (ACSM), which considers it essential to perform 150 min of moderate aerobic activity or 75 min of vigorous aerobic activity per week and at least 2 days of resistance training [13,17,20]. 

The relationship between physical activity and breast cancer has been demonstrated in several studies that analyzed and compared the effects of different exercise programs in breast cancer survivors, finding significant improvements in quality of life [21,22], physical function, and muscle strength [21,23]. More specifically, the study by Wiskemann et al. (2016) based on a 12-week resistance training program showed gains in muscle strength [24]. In several studies where the training programs combined endurance with aerobic exercise for 12 weeks, the improvements were significant in muscle strength, level of physical activity, and quality of life [21,25,26,27,28,29]. In addition, studies on mixed programs combining aerobic and strength exercises [30,31] showed important improvements in aerobic capacity, maximum oxygen consumption, muscle strength, reduction in the percentage of fat mass, and, above all, improved quality of life. Improvements were also found in physical, psychological, social, and quality of life parameters from training programs based on dragon boat rowing in women with breast cancer [18,32,33,34].

Women breast cancer survivors have found rowing to be an activity that improves the sequelae of the disease [35], such as reducing pain, increasing the range of movement in the upper limbs, improving muscle activation, and increasing strength and muscle function [36,37].

In this sense, rowing is considered one of the most complete water sports, involving the work of the musculature of both the upper and lower limbs [38] and almost all the body’s musculature [39]. It is a sport in which symmetrical movements are performed that do not require forced position and that combine the work of strength and aerobic endurance [18]. Several studies have shown that this type of activity improves the quality of life of cancer patients, including psychological, physical, social, and emotional aspects, favoring their rehabilitation, self-esteem, and normalizing their daily life [37,40,41].

In addition, the practice of rowing has psychosocial benefits for cancer survivors [37,42] because it is a team sport that promotes the development of social relationships, and they find the support they need in other women who have gone through or are going through the same situation. Additionally, rowing is an outdoor activity, which provides them with extra motivation to adhere to physical activity and improves their quality of life [41,43,44].

Finally, in studies using the short form of the International Physical Activity Questionnaire (IPAQ-SF) to examine changes in physical activity [28,29] and the Short Form 36 Health Survey (SF-36) for changes in quality of life [27,30] following physical activity interventions in breast cancer patients, it was found that activity levels increased, and the different domains of quality of life improved after completion of the training programs.

The purpose of the present longitudinal study is to determine the influence of a 12-week rowing training program on quality of life and physical activity levels in women who have survived breast cancer.

## 2. Materials and Methods

### 2.1. Design and Participants

The study, according to Hernández, Fernández, and Baptista (2014), is a non-experimental longitudinal panel design, given that the same participants have been measured or observed at all times or points in time [45].

Participants (*n* = 28) aged 52.3 ± 3.8 years were recruited under the condition of having overcome breast cancer. The women were diagnosed 4.7 ± 3.0 years earlier, had different stages of disease, and had undergone surgery, as shown in Table 1.

Breast cancer survivor (BCS) is the name given to women who have been diagnosed with breast cancer and have had to undergo surgery and chemotherapy and/or radiotherapy treatments.

To take part in this research, we searched various breast cancer associations in Malaga for women who wanted to do sports (rowing) and who met the following characteristics: having overcome breast cancer, having completed chemotherapy and radiotherapy treatments, and having the oncologist’s approval to do physical activity. No more than 10 years had passed since the cancer diagnosis. All of them were still taking tamoxifen.

The sample was selected based on compliance with these inclusion criteria.

The study was carried out at the RC Mediterráneo in Málaga involving women from the Sport Association Málaga D.B. Forty-eight participants were invited to take part: 10 initially withdrew due to compatibility/work/family and transport problems, and 10 were excluded because they did not attend 90% of the sessions.

After the initial selection, the nature of the study was explained to the participants, indicating that their anonymity would be maintained at all times, following the ethical considerations of the Sport and Exercise Science Research [46], as well as the principles included in the Declaration of Helsinki [47], which define the ethical guidelines for research in human subjects. The University of Malaga assigned the identification number 65-2020-H, which is registered with the Ethics Committee. The participants provided written informed consent, and throughout the intervention and afterwards, we acted under the provisions of the Organic Law 3/2018, of December 5, on the Protection of Personal Data and Guarantee of Digital Rights, regarding the protection of personal data under Spanish legislation. After signing the informed consent, the physical activity (IPAQ-SF) and the health-related quality of life (SF-36) questionnaires were administered.

The intervention lasted 12 uninterrupted weeks in which the women carried out two weekly sessions as described above. Both at the beginning and at the end of the program the participants were asked to complete the questionnaires.

### 2.2. Instruments

The participants also completed the short version of the International Physical Activity (IPAQ-SF) questionnaire to assess physical activity levels over the last 7 days. This questionnaire consists of seven questions that have acceptable measurement properties to monitor physical activity levels for adults aged 18 to 65 years in various settings, and it also reports the number of metabolic equivalents (METS) over the last 7 days [48]. Several studies have demonstrated the reliability of the IPAQ-SF for measuring the level of physical activity or the number of METS achieved during the last 7 days, obtaining similar results to other types of tests such as accelerometry or podometry [49,50,51].

The SF-36 Health Survey was used to assess health-related quality of life. This questionnaire consists of 36 items that report both positive and negative health status covering eight dimensions: physical function, social function, physical role, emotional role, mental health, vitality, bodily pain, and general health.

### 2.3. Intervention

Before starting the training program, participants were asked to complete the IPAQ-SF and SF-36 Health Survey questionnaires; in addition, they filled in the informed consent document to participate in the study.

The 12-week rowing training program was carried out at the RC Mediterráneo in Málaga, and it was divided into three parts of 4 weeks each. These stages progressively increased in intensity and were regulated through the participants’ subjective perception of effort using the Börg scale [52].

- Initial phase: mobility, proprioceptive, and postural control exercises. Main phase with rowing training. Final phase with stretching. Börg scale 5–6.
- Intermediate phase: mobility, proprioceptive, and postural control exercises. Main phase with rowing training. Final phase with stretching. Börg scale 6–7.
- Final phase: mobility, proprioceptive, and postural control exercises. Main phase with rowing training. Final phase with stretching. Börg scale 7–8.

Throughout the program, a weekly schedule was established consisting of three training days lasting 60–90 min per session. These sessions were supervised by a trainer who ensured attendance, correct execution of the tasks, and intensity of the sessions, in addition to excluding from the study those subjects who did not comply with at least 90% participation. All the exercises in these sessions were performed in a group. Exercises were generic and adjusted for people who have never rowed before. Each of the training sessions had the same structure:

1. Initial phase—performed with warm-up, mobility, proprioceptive, and postural control exercises; all exercises carried out in a multipurpose room (10–15 min).
2. Intermediate phase—performed in the Mediterranean Sea near the port of Malaga (Cruise Terminal/Malagueta Beach) using fixed-bench boats, typical of the Spanish Mediterranean, called Llauts, which are propelled by eight rowers and a coxswain or skipper [53], with each rower holding a single oar (40–60 min).
3. Final phase—flexibility exercises to relax the musculature and bring the body back to its initial state after exercise (10–15 min).

Both at the beginning and at the end of the program sessions were held to discuss issues, and the participants were asked to complete the questionnaires.

### 2.4. Data Analysis

All analyses were performed with IBM SPSS, version 25 (IBM Corp, Armonk, NY, USA). The significance level was defined as *p* < 0.05. The fit of the different variables to the normal distribution was assessed using graphic procedures and the Shapiro–Wilk test.

To examine the differences resulting from the rowing training performed by the participants, the medians of each variable pre- and post-intervention were analyzed using the Wilcoxon test for related samples (paired data). In addition, graphic analysis of the different variables was carried out using box-and-whisker plots. In addition, the effect size for the Wilcoxon test (r) was calculated by the Z-score [54]. In the Cohen’s guidelines for r, a large effect is defined as 0.5, a medium effect as 0.3, and a small effect as 0.1.

## 3. Results

Descriptive analyses of the different study variables (Table 2), level of physical activity, and quality of life are shown below, differentiating between pre-intervention and post-intervention values.

For the variables associated with engaging in physical activity obtained from the IPAQ-SF and the quality of life through the SF-36 questionnaire, the Wilcoxon test was carried out to determine whether significant differences exist between the pretest and posttest data.

**Figure 1:**
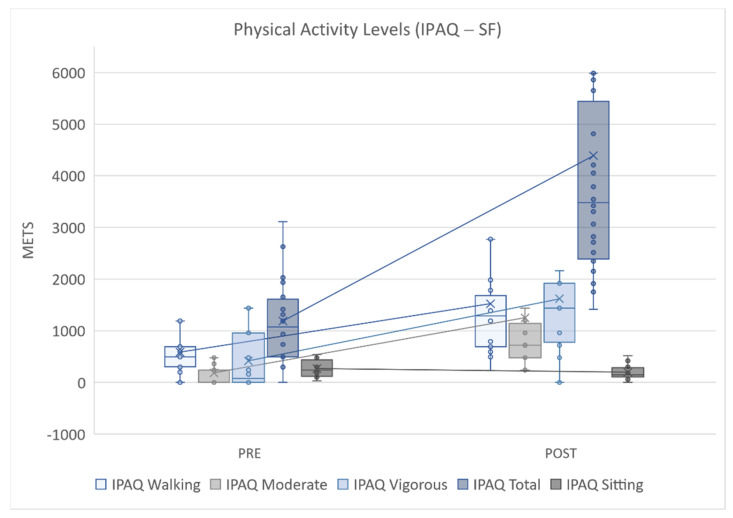
Physical activity levels (IPAQ-SF); PRE = pretest; POST = posttest; METS = metabolic equivalents.

Figure 1 depicts all physical activity variables, showing improvements after the intervention in levels of walking (Dif Mdn = 495 < 1287, z = −4.201, *p* = 0.000, r = 0.79), moderate (Dif Mdn = 0.00 < 720.00, z = −4.314, *p* = 0.000, r = 0.81), vigorous (Dif Mdn = 80.00 < 1440.00, z = −4.043, *p* = 0.000, r = 0.76), and total physical activity (Dif Mdn = 1075.50 < 3483.00, z = −4.286, *p* = 0.000, r = 0.81), all of which were significant. The lower value obtained for the IPAQ sitting variable (Dif Mdn = 240.00 > 150.00, z = −3.075, *p* = 0.002, r = 0.58) after the intervention compared to before the intervention indicates the participants were more active and spent less time sitting throughout the week. Regarding the effect size, the differences had a large effect and were statistically significant, as they presented values greater than 0.5.

**Figure 2:**
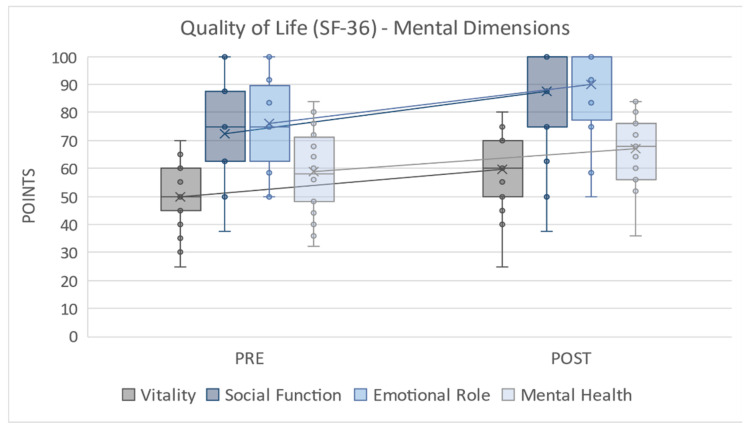
Quality of Life (SF-36)—Mental Dimensions; PRE = pretest; POST = posttest.

Regarding the variables associated with quality of life, Figure 2 displays the results for the mental dimensions, showing improvements after the intervention in vitality (Dif Mdn = 50.00 < 60.00, z = −2.879, *p* = 0.004, r = 0.54), social function (Dif Mdn = 75.00 < 100.00, z = −3.247, *p* = 0.001, r = 0.61), emotional role (Dif Mdn = 75.00 < 100.00, z = −3.268, *p* = 0.001, r = 0.61), and mental health (Dif Mdn = 58.00 < 68.00, z = −2.836, *p* = 0.005, r = 0.54), all of which were significant. As for the effect size, its values were relevant as they were above 0.5.

**Figure 3:**
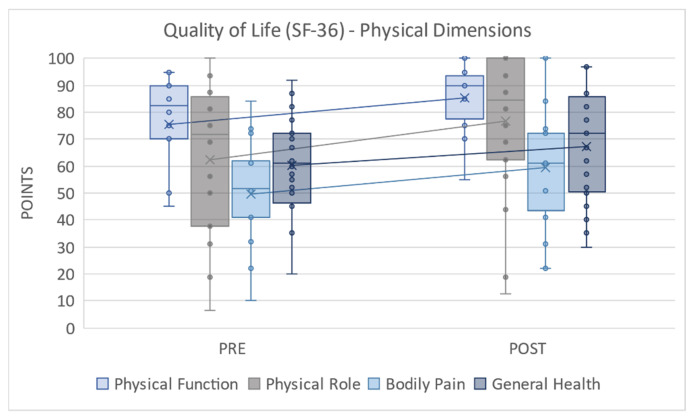
Quality of Life (SF-36)—Physical Dimensions; PRE = pretest; POST = posttest.

Focusing on the physical dimensions, Figure 3 shows that all variables improved significantly after the intervention, including general health (Dif Mdn = 61.00 < 72.00, z = −2.006, *p* = 0.045, r = 0.37). In the case of the variation in bodily pain (Dif Mdn = 51.50 < 61.00, z = −2.472, *p* = 0.013, r = 0.47), this may indicate that the participants reported less pain overall after the intervention. In other words, engaging in rowing decreases bodily pain in women who are breast cancer survivors or increases the pain threshold in the women studied. In addition, improvements after the intervention in physical role (Dif Mdn = 71.88 < 84.38, z = −3.866, *p* = 0.000, r = 0.73) and physical function (Dif Mdn = 82.50 < 90.00, z = −3.256, *p* = 0.001, r = 0.62) were shown, which indicates that the participants had fewer limitations when performing any physical activity compared to prior to the intervention. In terms of effect size, the data show that the differences were relevant, as they presented values between 0.3 and 0.5. This suggests that a 12-week rowing training program, adapted to women who have had breast cancer, helps to improve the perceived ability to perform other activities.

**Figure 4:**
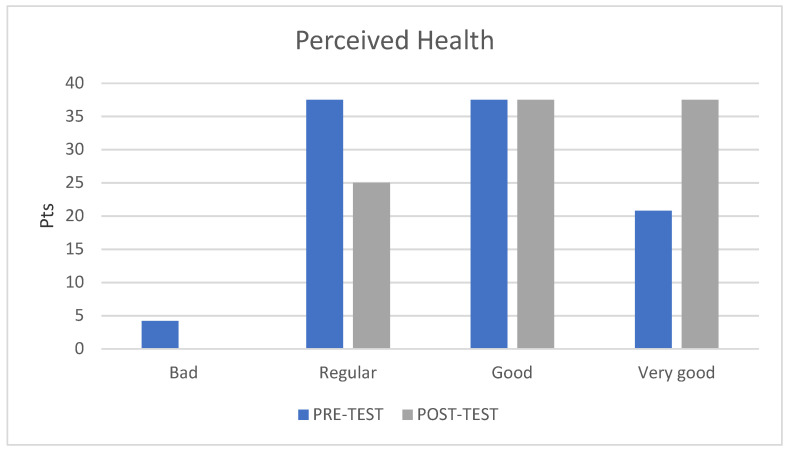
Variation in perceived health; Pts = points.

Finally, in terms of overall perceived health of the participants, a significant improvement was detected after the physical activity intervention (Figure 4). After completing the rowing program, a tendency towards an improved perception of health emerged: the number of women who reported having poor or fair health decreased, and those who claimed having “very good health” increased, with 75% of the participants reporting their health status to be good or very good.

In summary, after an intervention based on a 12-week rowing training program tailored to women who have had breast cancer, all variables showed significant improvements, including those concerning level of physical activity and quality of life. That is, the participants were more physically active and less sedentary throughout the week. In addition, their perceived physical, emotional, and mental health status improved, which indicates fewer limitations in terms of physical activity, social life, and vitality.

## 4. Discussion

To date, studies relating rowing to improvements in quality of life or in the level of physical activity in breast cancer survivors are practically non-existent. In this sense, some research can be found in which other types of sports, such as dragon boating [32,33], yoga, Pilates [55,56], or endurance, strength, or aerobic exercise training programs [22,24,31], report improvements at physical, psychological, and social levels in women breast cancer survivors.

More specifically, upon examining studies that used different types of physical activity programs, the 12-week resistance training program proposed by Wiskemann et al. (2016) reported improvements only in muscle strength [24]. Other programs that combined aerobic and strength exercises for 24 weeks led to improvements in muscle strength, aerobic capacity, and only some quality of life dimensions [31], while those that combined resistance and aerobic exercises for 16 weeks showed improvements in the quality of life and physical fitness of the participants [26]. Harris (2012) and McDonough et al. (2018) reported that dragon boat rowing [32,33] led to physical, psychological, and social improvements in women breast cancer survivors.

In our study, based on a rowing training program lasting only 12 weeks, improvements were observed in all levels of physical activity, as measured by the IPAQ-SF, including increased levels of walking, moderate and vigorous physical activity, and total physical activity as well as decreased sedentary activity of the participants; improvements were shown in all dimensions of quality of life. The results obtained in the present research are superior to those reported in previous studies; in addition, a shorter intervention time was required.

These results may be due to the fact that rowing is a sport that involves the muscles of both the lower and upper limbs and almost every muscle in the body [38]. In addition, rowing is a cyclic and symmetrical sport that combines overall strength with aerobic endurance [18]; it is based on cyclic and alternating movements of flexion/extension of the limbs and stabilization of the trunk and back muscles [57]. In this sense, the involvement of the whole body in physical activity, such as rowing, leads to improvements in quality of life and physical function as well as reduction in body fat in women breast cancer survivors [44,58,59].

In the study by Park et al. (2019), through a training program combining aerobic and resistance exercises, physical activity levels increased after the 12-week intervention, rising in 63,4% of the participants. At baseline, 33% of participants were inactive, 49.6% minimally active, and 17.4% health-enhancing physical activity (HEPA) active; after intervention the percentages improved, being 15.3%, 50.4%, and 34.2%, respectively [28]. It is of interest to compare these results with those of our study, which used an intervention of the same duration. In our study, overall physical activity levels increased in all participants after the rowing program with respect to the initial measurement. It should also be noted that prior to the intervention, about 50% of the participants reported no moderate or vigorous physical activity, whereas afterwards, all the participants reported engaging in moderate physical activity, and less than 10% reported no vigorous activity.

Regarding quality of life parameters, the study by Mascherini et al. (2020) shows improvements in physical function, social function, general health, and mental health after 6 months of training [60]. Di Blasio et al. (2017) after 12 weeks of intervention only found improvements in physical function, physical role, bodily pain, and general health [23]. Dolan et al. (2018) also showed that after a 22-week training program all SF-36 quality of life dimensions improved, with the exception of bodily pain [61].

Some of the limitations of this study reside in the scarcity of the sample. Although homogeneity was presented for the variables evaluated in this article, it would be interesting to know what the effects of this rowing program are from different times since the cancer was diagnosed or whether mastectomy is present or not.

The results of the present study demonstrate that rowing training has a greater influence on quality of life in breast cancer survivors than previously reported, as significant improvements were found in all quality of life parameters after an intervention of shorter duration. This implies that perceived physical, emotional, and mental health status and the perceived ability to perform other activities improved to a greater extent after our rowing-based training program. Indeed, 75% of the participants reported their health status as good or very good after the intervention, with a considerable increase compared to the initial data.

## 5. Conclusions

Previous studies have reported improved quality of life and reduced physical inactivity in female breast cancer survivors through training programs such as aerobic, strength, resistance, and dragon boat exercises. In the present study, we have shown that an intervention of just twelve weeks in length using rowing training tailored to women who have had breast cancer produced improvements in all levels of physical activity and reduced physical inactivity, with respect to the initial measurement. In addition, after the intervention, the physical, emotional, and mental health status of the participants improved, leading to fewer limitations in their daily routines, physical activity, social life, and vitality. Our rowing program showed greater benefits in health and quality of life than other studies of various durations. We can therefore conclude that rowing training contributes to increasing daily physical activity as well as improving health status and the different dimensions of quality of life in women who have survived breast cancer.

## Figures and Tables

**Table 1:** Characteristics of the breast cancer survivor sample.

Age (Years)	52.30 ± 3.78	
Years from Diagnosis	4.68 ± 3.00	
Stage (%)	1	4.54
	2	36.36
	3	54.54
	4	4.54
Surgery (%)	Preservation	56.53
	Total Mastectomy	43.47

**Table 2:** Descriptive analysis of the variables pretest and posttest.

Variables	Mean		Percentiles						Minimum		Maximum	
			25		50 (Mdn)		75					
	Pre	Post	Pre	Post	Pre	Post	Pre	Post	Pre	Post	Pre	Post
IPAQ Walking (METS)	587.8 ± 432.1	1522.1 ± 1353.1	305.3	693.0	495.0	1287.0	693.0	1683.0	0	231	1485	6930
IPAQ Moderate (METS)	185.0 ± 260.0	1250.0 ± 1664.2	0.0	480.0	0.0	720.0	240.0	1140.0	0	240	960	8400
IPAQ Vigorous (METS)	416.7 ± 513.1	1620.0 ± 1608.0	0.0	780.0	80.0	1440.0	960.0	1920.0	0	0	1440	7680
IPAQ Total (METS)	1189.5 ± 835.0	4392.1 ± 3341.7	495.0	2388.0	1075.5	3483.0	1611.0	5442.7	0	1413	3108	16290
IPAQ Sitting (METS)	267.8 ± 155.4	197.8 ± 135.2	120.0	104.3	240.0	150.0	435.0	285.0	30	0	540	520
Physical Function (Points)	75.4 ± 18.8	85.2 ± 13.3	70.0	77.5	82.5	90.0	90.0	93.8	30	50	95	100
Physical Role (Points)	62.5 ± 26.2	76.6 ± 25.4	37.5	62.5	71.9	84.4	85.9	100.0	6.3	12.5	100	100
Bodily Pain (Points)	49.7 ± 18.6	59.3 ± 22.2	41.0	43.5	51.5	61.0	62.0	72.0	10	22	84	100
General Health (Points)	60.1 ± 17.6	67.1 ± 19.9	46.3	50.5	61.0	72.0	72.0	85.8	20	30	92	97
Vitality (Points)	50.0 ± 12.9	59.8 ± 13.4	45.0	50.0	50.0	60.0	60.0	70.0	20	25	70	80
Social Function (Points)	72.4 ± 22.7	87.5 ± 18.4	62.5	75.0	75.0	100.0	87.5	100.0	12.5	37.5	100	100
Emotional Role (Points)	76.0 ± 15.8	89.9 ± 14.7	62.5	77.1	75.0	100.0	89.6	100.0	50	50	100	100
Mental Health (Points)	58.8 ± 13.9	67.0 ± 11.9	48.0	56.0	58.0	68.0	71.0	76.0	32	36	84	84

Pre = pretest; Post = posttest; Mdn = median IPAQ = International Physical Activity Questionnaire; METS = metabolic equivalents; 1 MET = 3.5 mL O_2_ × kg ^−1^ × min ^−1^.

## Data Availability

Data consent was obtained from all subjects involved in the study.
